# Ratio and difference of the cross-sectional area of median nerve to ulnar nerve in diagnosing carpal tunnel syndrome: a case control study

**DOI:** 10.1186/s12880-019-0351-3

**Published:** 2019-07-04

**Authors:** Yi-Wei Chang, Tsung-Cheng Hsieh, I-Shiang Tzeng, Valeria Chiu, Pei-Jung Huang, Yi-Shiung Horng

**Affiliations:** 1 Department of Physical Medicine and Rehabilitation Taipei Tzuchi Hospital, Buddhist Tzuchi Medical Foundation, No.289, Jianguo Rd., Xindian Dist, New Taipei City, 231 Taiwan, Republic of China; 2 Institute of Medical Sciences, Tzu Chi University, Hualien, Taiwan, Republic of China; 3 Department of Research Taipei Tzuchi Hospital, Buddhist Tzuchi Medical Foundation, New Taipei City, Taiwan, Republic of China; 4 Department of Medicine, Tzu Chi University, Hualien, Taiwan, Republic of China

**Keywords:** Carpal tunnel syndrome, Median nerve, Ulnar nerve, Ultrasound, Median nerve entrapment

## Abstract

**Background:**

To evaluate the diagnostic accuracy of the median-to-ulnar nerve ratio (MUR) and the median-to-ulnar nerve difference (MUD) in patients with carpal tunnel syndrome (CTS).

**Methods:**

In this study, 32 patients with CTS and 32 healthy volunteers were evaluated. All participants received a series of tests and ultrasound examination for the evaluation of the following criteria: cross-sectional area of the median nerve at the pisiform level (CSA-P), swelling ratio (SR), MUR, MUD, and flattening ratio (FR).

**Results:**

CSA-P, SR, MUR, and MUD were all significantly larger in the patients with CTS than in the healthy volunteers. The areas under the receiver operator characteristic curves of MUD, MUR, CSA-P, and SR were 0.78, 0.75, 0.70, and 0.61 respectively. MUD had higher sensitivity (84%) than MUR, CSA-P, and SR (sensitivity: 63, 63, and 53%, respectively).

**Conclusions:**

By using the ulnar nerve area at the pisiform level as an internal control parameter, the MUD and MUR methods showed higher diagnostic accuracy than SR in patients with CTS. Further application of these methods in research and clinical settings is recommended.

**Trial registration:**

Clinicaltrial.gov NCT03033173. Registered 18 January 2017. Retrospectively registered.

## Background

Carpal tunnel syndrome (CTS) is a common compressive neuropathy of the median nerve at the wrist level; it accounts for 90% of entrapment neuropathy cases. Its prevalence rate is nearly 3.8% in the general population. Its peak age ranges from 40 to 60 years, and it is predominant in women [1].

Traditionally, patients are initially assessed for clinical symptoms such as numbness, tingling, or hyperesthesia over the median nerve innervating digits. Symptoms might aggravate nocturnally or be induced after prolonged wrist flexion or extension. Provocative tests, such as Phalen’s test or Tinel’s test, may also be performed to detect any subtle irritable neuropathic symptoms. In severe cases, thenar muscle atrophy might be evident [2, 3]. However, in mild cases, patients might only present vague symptoms or less specific clinical signs. Therefore, a nerve conduction study (NCS) is used to provide more objective evidence for diagnosing CTS. According to the summary statement of electrodiagnostic studies of CTS issued by the American Association of Neuromuscular & Electrodiagnostic Medicine, sensory and motor NCSs of the median nerve can provide a high specificity rate (98%) and an optimal sensitivity rate (> 62%) [4]. A more recent study showed that a short-segment, onset latency-based NCS can provide an improved sensitivity rate of 75% [5]. However, these data imply that there is still a certain degree of false negative rates (10–25%) in clinical evidence of CTS [6, 7]. Hence, imaging studies, such as ultrasonography (US) and magnetic resonance imaging (MRI), of the median nerve at the wrist level were introduced to provide an objective evaluation of median nerve morphology [8, 9].

Compared with NCSs, imaging studies have the advantage of causing less discomfort and can detect structural abnormalities inside the carpal tunnel. US is more advantageous than MRI because of its portability and cost effectiveness. Furthermore, US is less time consuming, can provide dynamic evaluation, and can guide possible further interventional treatment, including carpal tunnel median nerve injection [10].

In the US-related criteria for CTS, the cross-sectional area (CSA) method, which involves the measurement of median nerve CSA at the pisiform level (carpal tunnel inlet), is regarded as the most accurate method for diagnosing CTS [11–13]. However, using a single cut-off value to diagnose CTS may underestimate the prevalence of CTS in a study population, given the variability of the median nerve among individuals with varying body weight, age, and sex [14, 15]. Therefore, using CSA of the proximal median nerve at the forearm level as an internal control parameter has previously been proposed. The ratio of the CSA of the median nerve at the pisiform level to that of the median nerve at the forearm level is defined as the swelling ratio (SR) [10, 11] or the wrist-to-forearm nerve ratio (WFR) [6].

Relative to the CSA method, the comparative method of WFR has shown inconsistent results in previous studies. One study showed that the WFR method had higher sensitivity than the CSA method [6], whereas another study reported no significantly higher diagnostic ability for WFR [16]. Moreover, two studies have shown that median nerve CSA at the forearm level is significantly smaller in patients with CTS than in healthy controls [6, 17]. However, other studies have not provided such a finding [8, 18]. The inconsistent results potentially affect the reliability of forearm median nerve CSA as an internal control. On the contrary, the ulnar nerve at the wrist level has been shown to have no significant difference in CSA between patients with CTS and healthy controls [19]; accordingly, the ulnar nerve is suitable to serve as an alternative internal control to compare with the median nerve. Previous studies tried to use median-to-ulnar-nerve ratio (MUR) to compare the CSAs of median to ulnar nerves at wrist level; however, their results did not provide superior diagnostic value comparing to the CSA method [20, 21]. Therefore, in this study, we propose using the difference between the CSAs of the median and ulnar nerves at the pisiform level as a new diagnostic criterion, i.e., median-to-ulnar-nerve difference (MUD). The aim of this study is to compare the diagnostic accuracy of the MUR and the MUD in patients with CTS.

## Methods

### Patients

Patients with CTS were recruited from the physical medicine and rehabilitation department of a community hospital. Control participants were recruited from the hospital staff, healthy volunteers, and their friends. This study was conducted with the approval of our Institutional Review Board (Approval No. 01-XD15–036). Informed consent was obtained from all participants prior to the study.

The patients with CTS were required to have subjective symptoms of numbness, tingling pain, or dysesthesia over the median nerve-innervated area of the involved hand. Furthermore, the patients were required to have positive responses in either Tinel’s test or Phalen’s test during physical examination and were required to show evidence of median neuropathy at the wrist level in the NCS. Control participants must not exhibit symptoms or signs of CTS and have no abnormal findings in the electrodiagnostic study of the median and the ulnar nerves across the wrist joint. The exclusion criteria for the patients with CTS and control participants were as follows: (A) age < 20 years; (B) electrodiagnostic evidence of ulnar neuropathy at the wrist level; (C) previous trauma or surgical history of the hand; (D) medical history of hypothyroidism, diabetes mellitus, uremia, rheumatoid arthritis, amyloidosis, and acromegaly; and (E) pregnancy.

All participants received a series of examination, including baseline survey, physical examination, NCS, and ultrasound examination. Physical examination included the grip strength test, the Semmes–Weinstein monofilament sensory test, and Tinel’s test and Phalen’s test. In addition, each participant completed the Boston CTS questionnaire.

### Physical examination

During Phalen’s test, the participants were asked to hold both wrists in the full flexion position for 1 min. The participants experiencing characteristic CTS symptoms (tingling, numbness, and dysesthesia sensation in the median nerve-innervated area) were considered to exhibit positive responses. A positive result for Tinel’s test was defined as elicitation of characteristic CTS symptoms when the median nerve was tapped as it passed through the carpal tunnel [22]. Grip strength was measured using a handheld dynamometer; in the grip strength test, the participant forcefully flexed all the finger joints in the sitting position with elbow flexion of approximately 90° and the forearm and wrist in a neutral position. This procedure was repeated three times, and the mean score was calculated [23].

The Semmes–Weinstein monofilament sensory test was applied on the finger tips and hand with the wrist in a neutral position. A force-calibrated nylon filament was pressed to the skin, and a gradually increasing force was applied until the filament bent to form a “C.” The response of the participants was considered positive if they could identify which digit was tested with their eyes closed. A weighted score from 1 to 5 was estimated depending on the calculated force of each filament (a lower score indicated greater force) [24].

### Boston CTS questionnaire

The Boston CTS questionnaire is self-administered and is composed of two parts: the symptom severity scale (11 questions) and the functional status scale (8 questions). All questions are scored from 1 to 5 depending on the participants’ symptoms (1 indicates no symptom, and 5 indicates the most severe symptom). Finally, the average of the symptom severity score and the functional status score is calculated. The reproducibility of the internal consistency of the questionnaire has been validated in a previous study [24].

### Nerve conduction study

In this study, an NCS was performed using Neuropack M1 MEB-9200 J/K electrodiagnostic equipment (Nihon Kohden Corporation, Tokyo, Japan). Each participant was tested in the supine position in a quiet, air-conditioned room with room temperature maintained at 26 °C. The temperature of each hand was maintained at ≥32 °C. The NCS was conducted using the supramaximal stimulation technique, in which a constant current stimulator was used and surface recording was performed [25]. Distal motor latencies of the median and ulnar nerve were respectively measured by placing a stimulating electrode at the wrist and a recording electrode 8 cm away from the stimulating electrode on the abductor pollicis brevis muscle and the adductor digiti minimi muscle. For sensory NCSs of the median and ulnar nerves, a standard distance of 14 cm was maintained between the stimulating electrode at the wrist and the recording electrodes at the ring finger on radial and ulnar sides [4].

The diagnosis of CTS was established if one of the two following criteria was met in the median nerve NCS: distal motor latency > 4.4 ms and distal sensory latency > 3.4 ms [26]. The participants were screened and excluded for subclinical ulnar neuropathy at the wrist level on the basis of the following criteria: ulnar nerve NCS distal motor latency > 3.6 ms and ulnar nerve NCS distal sensory latency > 3.4 ms [27, 28].

### Ultrasound examination

Ultrasound examination was conducted using the GE logic P6 device (General Electric Medical Systems, Milwaukee, WI, USA) with an 11 L linear array transducer (12 MHz). The participants were asked to lie on the bed in the supine position with the elbow extended, forearm supinated, wrist in the neutral position, and fingers semiflexed. The transducer was placed perpendicular to the nerve under examination using a transverse scanning technique. Care was taken to avoid applying additional forces on the transducer to prevent further nerve deformation.

The median nerve was identified as an oval structure surrounded by the hyperechoic epineurium at the pisiform level of the carpal tunnel, lying inferior to the flexor retinaculum. The distal wrist crease was used as the external landmark. The ulnar nerve was also identified at this level between the ulnar artery and rounded ventral aspect of the pisiform, which is normally smaller than the median nerve [29].

**Fig. 1:**
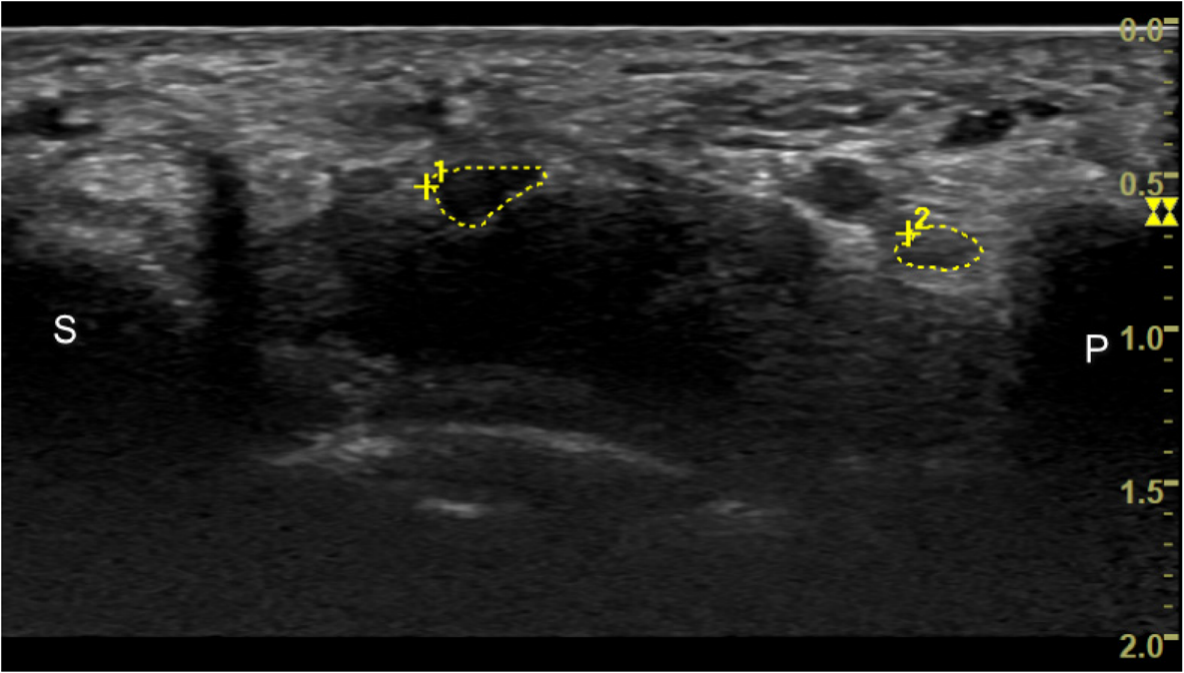
Cross-sectional area of the median nerve (large encircled area) and the ulnar nerve (small encircled area) at the pisiform level; P: pisiform bone; S: scaphoid bone

**Fig. 2:**
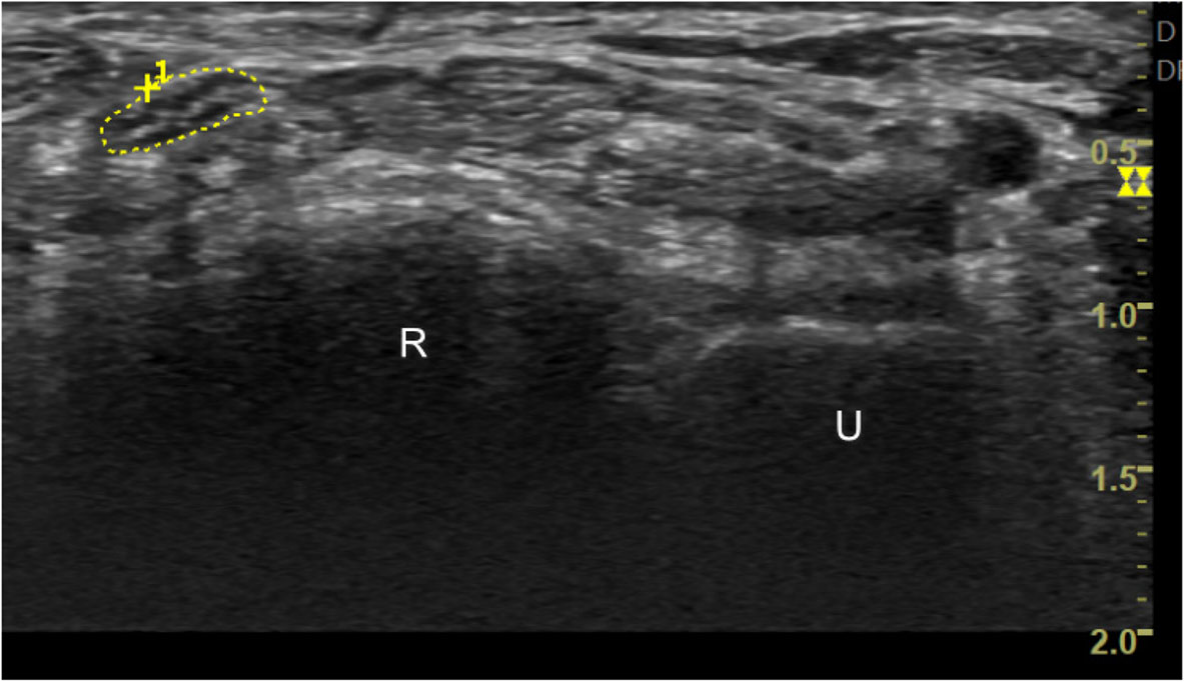
Median nerve cross sectional area at the distal radioulnar joint level (U: Ulnar bone; R: Radius bone)

**Fig. 3:**
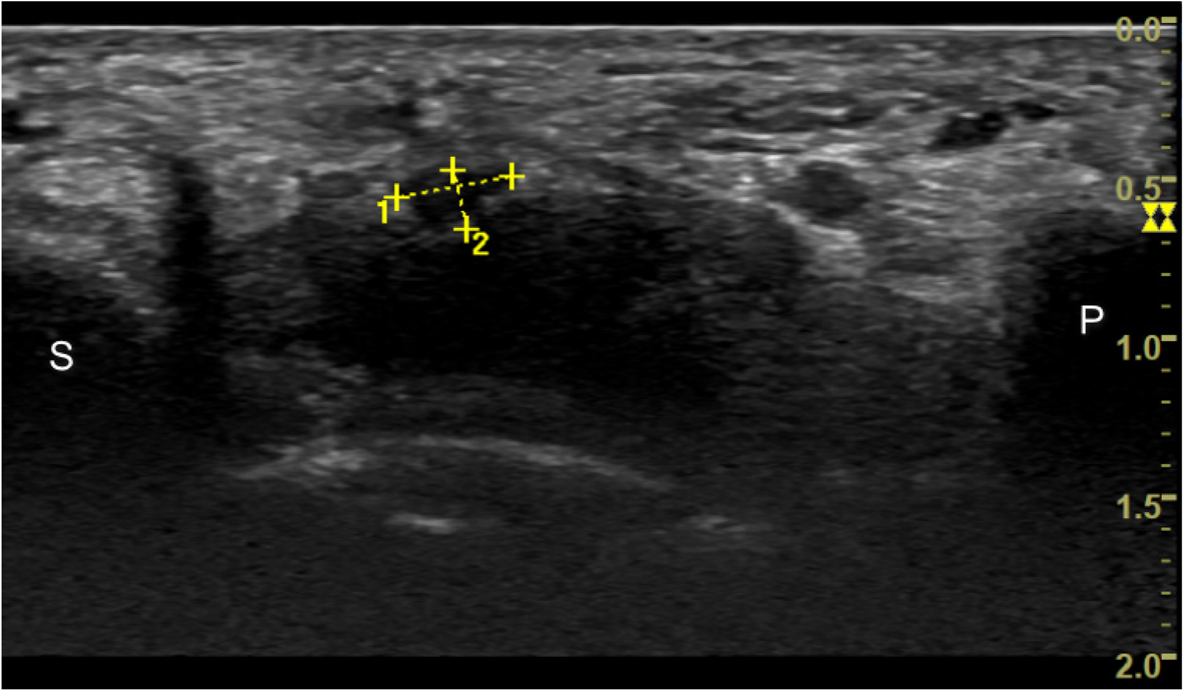
Long axis and short axis of median nerve at the pisiforms level (P: pisiform bone; S: scaphoid bone)

For CSA measurement, the inner border of the epineurium rim of each nerve was continuously traced. MUR is the ratio of the CSA of the median nerve to that of the ulnar nerve at the pisiform level, and MUD is the difference between the CSA of the median and ulnar nerves (Fig. 1). Median nerve CSA at the distal radioulnar joint (DRUJ) level was also acquired for estimating SR, which was defined as the ratio of the CSA of the median nerve at the pisiform level to the CSA of the median nerve at the DRUJ level. The DRUJ level could be identified between the distal radius and ulna, while tracing proximally from the pisiform level. (Fig. 2). The flattening ratio (FR) was calculated by dividing the length of the long axis by that of the short axis of the median nerve at the pisiform level (Fig. 3). Ultrasound images were acquired by a single investigator, who is board certified in ultrasound examination and has more than 5 years of experience in ultrasound examination. This investigator was blinded to the results of physical examination and NCS and the patients’ clinical status.

### Statistical analysis

Statistical analysis was performed using SAS Version 9.2 (SAS institute Inc., Cary, NC, USA). Data are presented as mean ± standard deviation. Student *t* and chi-squared tests were used to compare baseline characteristics between the two groups. Data derived from physical examination, NCS, and ultrasound examination were compared between the CTS and control groups. Each hand of the participants was individually analyzed. Both hands of the same participant were included when the patient had bilateral CTS or normal results were obtained for the control in the bilateral NCS. Considering a correlation between hands, the generalized estimate equation (GEE) was used after adjustment for the covariates of age, sex, and body mass index (BMI). Either the left or right hand of the participants was randomly selected for receiver operator characteristic (ROC) analysis. The ROC method uses a binary classifier system that plots the true positive rate (sensitivity) against the false-positive rate (1 − specificity). Youden’s index was used to obtain the optimal diagnostic point of each ultrasound diagnostic method. The diagnostic accuracy of sonographic criteria was measured by calculating the area under the ROC curve (AUC) [30].

## Results

Thirty-seven patients with CTS were initially screened. Among them, five patients were excluded because of subclinical ulnar neuropathy noted in the NCS. In total, 32 patients (56 hands) were included in the CTS group. Moreover, 37 healthy volunteers were screened; after asymptomatic hands with abnormal NCS findings were excluded, 32 healthy participants (59 hands) were included in the control group.

### Patient characteristics and Boston CTS questionnaire

The patients with CTS and healthy volunteers did not significantly differ in age, sex, marital status, smoking habit, dominant hand, and education level (Table 1). BMI was significantly higher in the patients with CTS (*P* = 0.01) than in the healthy volunteers. The symptoms severity score and functional status score were significantly higher in the patients with CTS (*P* < 0.0001) than in the healthy volunteers.

**Table 1:** Frequency distribution of demographic data for patients with CTS and healthy volunteers

	CTS patients *N* = 32 *N* (%)	Healthy volunteers *N* = 32 *N* (%)	*P* value^*^
Personal Characteristics			
Age(y)	52.0 (32.8–71.1)	5 50.1 (30.0–70.2)	0.45
Female	27 (84.4)	27 (84.4)	1.00
Married	26 (81.3)	22 (73.3)	0.46
Employed	15 (46.9)	25 (78.1)	0.01
Smoking habit	2 (6.3)	1 (3.1)	1.00
Right-hand dominant	32 (100.0)	31 (96.9)	1.00
Unilateral hand involved	7 (22)		
BMI (kg/m^2^)	24.4 (22.9–26.0)	22 2 22.1 (16.6–27.5)	0.01
Educational Level			0.014
College/University	7 (21.9)	17 (53.2)	
Senior High	14 (43.8)	12 (37.5)	
Junior High or below	11 (34.3)	3 (9.4)	
Boston CTS questionnaire			
Symptom severity score	2.2 (1.3–3.8)	1.0 (1.0–1.8)	< 0.0001
Functional status score	1.5 (1.0–3.1)	1.0 (1.0–1.1)	< 0.0001

Abbreviations: *BMI* body mass index, *CTS* carpal tunnel syndrome

^*^t test or chi square test

### Physical examination and NCS

The patients with CTS had significantly lower scores in the monofilament sensory test than did the healthy volunteers. Grip strength did not significantly differ between the two groups. The NCS revealed that the patients with CTS had significantly slower sensory nerve conduction velocity and longer sensory and motor distal latencies (Table 2).

**Table 2:** Comparison of results of physical examinations and NCS between hands with CTS and healthy hands

	CTS hands (*n* = 56)	Healthy hands (*n* = 59)	*P* value^*^
	Mean (SD)	Mean (SD)	
Monofilament sensory test	32.2 (2.7)	33.4 (1.7)	0.048
Grasp power (lb)	49.6 (19.7)	52.6 (16.3)	0.31
SNCV of median nerve (m/sec)	38.3 (9.1)	52.2 (6.5)	< 0.0001
Distal sensory latency of median nerve (ms)	3.9 (0.9)	2.7 (0.3)	< 0.0001
Distal motor latency of median nerve (ms)	4.9 (1.3)	3.5 (0.3)	< 0.0001

Abbreviations: *CTS* carpal tunnel syndrome, *NCS* nerve conduction study, *lb*. pound, *SNCV* sensory nerve conduction velocity, *m/sec* meter per second, *ms* milliseconds

^*^: comparison of differences between groups after adjusting for age, gender and body mass index (generalized estimating equation)

### Ultrasound examination

Median nerve CSA at the pisiform level, SR, MUR, MUD, and median nerve CSA at the DRUJ level were all significantly larger in the patients with CTS than in the healthy volunteers. Median nerve CSA was larger than ulnar nerve CSA in both groups. No significant difference was observed in the FR of median nerve and ulnar nerve CSAs at the pisiform level in both groups (Table 3). The ROC analysis of the four aforementioned diagnostic criteria revealed that MUD had the highest AUC of 0.78, with a clear cut-off value of 5.53 (sensitivity: 84%, specificity: 69%). The MUR method provided sensitivity of 63% and specificity of 84%, with a cut-off value of 3.28 and an AUC of 0.75. SR of the median nerve had a relatively low AUC of 0.61 and a cut-off value of 1.32 (sensitivity: 53%, specificity: 72%) (Table 4).

**Table 3:** Comparison of sonographical findings between hands with CTS and healthy hands

	CTS patients (hands =56)	healthy volunteer (hands =59)	*P* value^*^
	Mean (SD)	Mean (SD)	
CSA-P of median nerve (mm^2^)	11.3 (4.4)	8.8 (2.2)	< 0.0001
CSA-DRUJ of median nerve (mm^2^)	8.2 (2.9)	7.4 (1.7)	0.04
CSA-P of ulnar nerve (mm^2^)	3.1 (1.1)	3.5 (1.4)	0.08
Flattening ratio of median nerve	2.5 (0.7)	2.6 (0.9)	0.64
Swelling ratio	1.5 (0.9)	1.2 (0.3)	0.04
MUR	4.1 (2.3)	2.9 (1.5)	0.0206
MUD	8.2 (4.3)	5.3 (2.0)	< 0.0001

Abbreviations: *CTS* carpal tunnel syndrome, *CSA-P* cross-sectional area at the pisiform level, *CSA-DRUJ* cross-sectional area at the distal radioulnar joint level, *MUR* median-to-ulnar nerve ratio, *MUD* median-to-ulnar nerve difference

^*^: comparison of differences between groups after adjusting for age, gender and body mass index (generalized estimating equation)

**Table 4:** ROC analysis of CTS sonographical diagnostic criteria

	AUC (95% CI)	Cut-off value	Sensitivity (%)	Specificity (%)
CSA-P of median nerve (mm^2^)	0.70 (0.57–0.83)	10.35	63	84
SR	0.61 (0.47–0.75)	1.32	53	72
MUR	0.75 (0.63–0.88)	3.28	63	84
MUD	0.78 (0.67–0.90)	5.53	84	69

Abbreviations: *ROC* receiver operating characteristic, *AUC* area under the curve, *CI* confidence interval, *CSA-P* cross-sectional area at the pisiform level, *SR* swelling ratio, *MUR* median nerve to ulnar nerve ratio, *MUD* median-to-ulnar nerve difference

### Discussion

Our data showed that by using the CSA of the ulnar nerve at the pisiform level as an internal control parameter, MUD and MUR provided higher diagnostic accuracy than SR in the patients with CTS (Table 4). Despite similar AUC, MUD had higher sensitivity (84%) than MUR (63%).

Regarding the application of US for diagnosing CTS, Buchberger et al. first addressed the following four typical ultrasonographic presentations in patients with CTS: A) a flattening of the median nerve at the hamate level; B) an increase in the CSA of the median nerve at the pisiform level; C) an increase in the ratio of median nerve CSA at the pisiform level to median nerve CSA at the distal radius level; and D) palmar bowing of the transverse carpal ligament [31]. These presentations evolved into various ultrasonographic diagnostic criteria for CTS, such as the FR (the ratio of the length of the long axis of the median nerve to that of the short axis of the median nerve), CSA, and SR methods [11, 32, 33].

The FR method was later examined in several clinical studies, which have provided controversial results. Using FR > 3.3 as the diagnostic criterion, Duncan et al. compared FR between patients with CTS and normal participants, and they indicated a low likelihood ratio of 1.53 [34]. Mallouhi et al. used FR > 3 as the cut-off value and obtained sensitivity of 60% and specificity of 76% [35]. One recent study showed that FR does not significantly differ between symptomatic and asymptomatic groups and shows no correlation to electrodiagnostic testing [18]. Consistent with these findings, FR in our study did not significantly differ between the healthy volunteers and patients with CTS.

The CSA method initially showed suboptimal accuracy compared with the other methods, possibly because of indirect measurement by assuming that the entire median nerve CSA is elliptical in shape [(long axis × short axis × π)/4] [31, 36]. Sensitivity of the CSA method improved after a direct measurement method (continuous tracing of the inner border of the endoneurium) was used, and the CSA method was correlated with electrodiagnostic studies [11–13]. Our study revealed that median nerve CSA at the pisiform level significantly differed between the healthy volunteers and patients with CTS, and it provided fair accuracy when 10.35 mm^2^ was used as the diagnostic value.

The use of one definitive cut-off value of median nerve CSA to diagnose CTS has been questioned for various reasons. First, CSA may be affected by several demographic factors, such as body height, body weight, age, and sex [14, 37]. According to one study, healthy volunteers with higher body height, higher body weight, and male sex have larger nerve CSA in the upper limb nerves (median nerve, ulnar nerve, radial nerve, and musculocutaneous nerve) [38]. Zaidman et al. also showed that with an increase of 1 cm in the body height of a healthy volunteer, median nerve CSA increased by 0.06 mm ^2,^ [37]. Second, nerve CSA might differ among ethnicities. For example, some studies have demonstrated that the mean median nerve CSA of the American population is larger than that of the Korean population [14, 38].

Therefore, a comparison of the ratio between two levels of the median nerve, such as in the SR method, was introduced to avoid potential bias caused by the aforementioned individual variations. The SR method compares median nerve CSA between the pisiform level and radioulnar joint level. This method has been reported to have sensitivity of 66.7% for diagnosing clinical symptoms in patients with CTS [10]. However, for the SR method, our study revealed relatively low sensitivity (53%) and AUC (0.61) when ≥1.32 was used as the cut-off value. The SR method also has limitations for detecting more severe CTS [39]. This might be explained by the phenomenon of concurrent swelling of the median nerve proximal to the entrapment site. As previously reported, proximal swelling of the median nerve beyond the pisiform level was noted in patients with moderate CTS [40], which may lead to a reduction in median nerve CSA differences between the pisiform level and the DRUJ level. Consequently, it might undermine the diagnostic accuracy of the SR method. This phenomenon of concurrent proximal swelling of the median nerve was also noted in our study; as shown in Table 3, CSAs of the median nerve were significantly larger in the patients with CTS than in the healthy volunteers, not only at the pisiform level but also at the DRUJ level.

The WFR method compares the median nerve at the pisiform level to the forearm level, ranging from 4 to 12 cm proximal to the distal wrist crease according to different studies [41]. Although attempts have been made to exclude the potential proximal swelling of the median nerve at the DRUJ level in this method, a significant difference in median nerve CSA at the forearm level between patients with CTS and healthy control has still been noted in previous studies^6,17^, thus undermining the role of the forearm median nerve as an internal control [6, 17]. Furthermore, dense connective tissues are present around the median nerve at the forearm, which occasionally renders identification of the true boundary of the epineurium challenging [42].

Using ulnar nerve CSA at the pisiform level as an internal control and comparing it with median nerve CSA can avoid the confounding effects of proximal median nerve swelling. Eom et al. showed that there was no change in ulnar nerve CSA between patients with CTS and healthy controls; they also demonstrated a strong correlation between MUR and the electrophysiologic stage of CTS [19]. Furthermore, Yurdakul et al. demonstrated that compared with the CSA and SR methods, the MUR method was the only method that positively correlated with symptom duration [21]. Based on these results, the ulnar nerve may be a suitable internal control for evaluating CTS. However, in previous studies, the MUR method did not show superior diagnostic value to the CSA method [20, 21]. In our study, the MUR method showed slightly higher AUC than the CSA method only (0.75 vs 0.70). Therefore, we introduced the MUD method, which compared the difference between the CSA of the median nerve and the CSA of the ulnar nerve at the pisiform level, as an alternative parameter to assist in diagnosing CTS. Klauser et al. introduced the wrist-to-forearm nerve difference (WFD), which is the difference between the CSAs of the median nerve at the carpal tunnel and forearm level [41]. It showed higher sensitivity and specificity than the CSA method. Compared with the WFR method, WFD also had a stronger correlation with CTS patients’ clinical severity despite similar AUC [43]. Our results also revealed the similar AUC of MUD and MUR (0.78 vs 0.75). However, MUD had the highest sensitivity (84%) compared with MUR, median nerve CSA at the pisiform level, and SR (sensitivity: 63, 63, 53%, respectively); thus, MUD is useful in screening CTS using US.

This study has some limitations that should be considered. First, we only recruited patients who met the clinical criteria of idiopathic CTS. This might not reflect the reality of daily clinical practice, where patients may have median neuropathy at the wrist level that may be secondary to various types of underlying conditions. Second, the inclusion criteria of this study included positive results of Tinel’s or Phalen’s test, which have moderate sensitivity. Moreover, we did not use the mixed-nerve short-segment comparative test as a diagnostic criterion in the NCS. Thus, the patients we recruited were more severely affected, and 59% of the patients had moderate to severe CTS (41% of these patients had slow sensory nerve conduction velocity and prolonged distal motor latency, and sensory or motor responses were absent in 18% of the patients). Therefore, further study may be required to explore the sensitivity and specificity of the MUR method in patients with CTS who exhibit mild symptoms. Third, the MUR method was based on the assumption that the ulnar nerve at wrist level is less likely to have compressive neuropathy; hence, it could be used as an internal control. Consequently, this method cannot be applied to patients with clinical symptoms of ulnar neuropathy at the wrist level.

## Conclusions

By using the cross-sectional area of ulnar nerve at the pisiform level as an internal control parameter, the MUD and MUR methods showed higher diagnostic accuracy than SR in patients with CTS. These methods can be used as alternative ultrasound diagnostic methods, and MUD has higher sensitivity than MUR. Further application of these methods in research and clinical settings is recommended.

## Data Availability

All the data needed to achieve the conclusion are contained within the paper. The raw data cannot be shared publicly due to ethical reason.

## Abbreviations

AUC: Area under the curve
BMI: Body mass index
CSA: Cross-sectional area
CTS: Carpal tunnel syndrome
DRUJ: Distal radioulnar joint
FR: Flattening ratio
GEE: Generalized estimate equation
MRI: Magnetic resonance imaging
MUD: median-to-ulnar-nerve difference
MUR: Median to ulnar nerve ratio
NCS: Nerve conduction study
ROC: Receiver operator characteristic
SR: Swelling ratio
US: Ultrasonography
WFR: wrist-to-forearm nerve ratio

## References

[1] Ibrahim I, Khan W, Goddard N, Smitham P (2012). Suppl 1: carpal tunnel syndrome: a review of the recent literature. Open Orthop J.

[2] Uchiyama S, Itsubo T, Nakamura K, Kato H, Yasutomi T, Momose T (2010). Current concepts of carpal tunnel syndrome: pathophysiology, treatment, and evaluation. J Orthop Sci.

[3] MacDermid JC, Doherty T (2004). Clinical and electrodiagnostic testing of carpal tunnel syndrome: a narrative review. J Orthop Sports Phys Ther.

[4] Jablecki C, Andary M, Floeter M, Miller R, Quartly C, Vennix M, Wilson J (2002). Practice parameter: electrodiagnostic studies in carpal tunnel syndrome report of the American Association of Electrodiagnostic Medicine, American Academy of Neurology, and the American Academy of physical medicine and rehabilitation. Neurology.

[5] Lew HL, Date ES, Pan SS, Wu P, Ware PF, Kingery WS (2005). Sensitivity, specificity, and variability of nerve conduction velocity measurements in carpal tunnel syndrome. Arch Phys Med Rehabil.

[6] Hobson-Webb LD, Massey JM, Juel VC, Sanders DB (2008). The ultrasonographic wrist-to-forearm median nerve area ratio in carpal tunnel syndrome. Clin Neurophysiol.

[7] Redmond MD, Rivner MH (1988). False positive electrodiagnostic tests in carpal tunnel syndrome. Muscle Nerve.

[8] Hunderfund ANL, Boon AJ, Mandrekar JN, Sorenson EJ (2011). Sonography in carpal tunnel syndrome. Muscle Nerve.

[9] Horng Y-S, Chang H-C, Lin K-E, Guo Y-L, Liu D-H, Wang J-D (2012). Accuracy of ultrasonography and magnetic resonance imaging in diagnosing carpal tunnel syndrome using rest and grasp positions of the hands. J Hand Surg.

[10] Keberle M, Jenett M, Kenn W, Reiners K, Peter M, Haerten R, Hahn D (2000). Technical advances in ultrasound and MR imaging of carpal tunnel syndrome. Eur Radiol.

[11] Roll SC, Case-Smith J, Evans KD (2011). Diagnostic accuracy of ultrasonography vs. electromyography in carpal tunnel syndrome: a systematic review of literature. Ultrasound Med Biol.

[12] Tai T-W, Wu C-Y, Su F-C, Chern T-C, Jou I-M (2012). Ultrasonography for diagnosing carpal tunnel syndrome: a meta-analysis of diagnostic test accuracy. Ultrasound Med Biol.

[13] McDonagh C, Alexander M, Kane D. The role of ultrasound in the diagnosis and management of carpal tunnel syndrome: a new paradigm. Rheumatology. 2014:keu275. 10.1093/rheumatology/keu275 25118315

[14] Cartwright MS, Shin HW, Passmore LV, Walker FO (2009). Ultrasonographic reference values for assessing the normal median nerve in adults. J Neuroimaging.

[15] Cartwright MS, Shin HW, Passmore LV, Walker FO (2007). Ultrasonographic findings of the normal ulnar nerve in adults. Arch Phys Med Rehabil.

[16] Visser LH, Smidt MH, Lee ML (2008). Diagnostic value of wrist median nerve cross sectional area versus wrist-to-forearm ratio in carpal tunnel syndrome. Clin Neurophysiol.

[17] Mhoon JT, Juel VC, Hobson-Webb LD (2012). Median nerve ultrasound as a screening tool in carpal tunnel syndrome: correlation of cross-sectional area measures with electrodiagnostic abnormality. Muscle Nerve.

[18] Roll SC, Evans KD, Li X, Freimer M, Sommerich CM (2011). Screening for carpal tunnel syndrome using sonography. J Ultrasound Med.

[19] Eom YI, Choi MH, Kim YK, Joo IS (2015). Sonographic findings in the ulnar nerve according to the electrophysiologic stage of carpal tunnel syndrome. J Ultrasound Med.

[20] ATAN T, GÜNENDİ Z (2018). Diagnostic utility of the sonographic median to ulnar nerve cross-sectional area ratio in carpal tunnel syndrome. Turkish J Med Sci.

[21] Yurdakul OV, Mesci N, Çetinkaya Y, Geler Külcü D (2016). Diagnostic significance of ultrasonographic measurements and median-ulnar ratio in carpal tunnel syndrome: correlation with nerve conduction studies. J Clin Neurol.

[22] Braddom RL. Physical medicine and rehabilitation: Elsevier Health Sciences; 2010.https://www.elsevier.com/books/physical-medicine-and-rehabilitation/braddom/978-1-4377-0884-4

[23] Brininger TL, Rogers JC, Holm MB, Baker NA, Li Z-M, Goitz RJ (2007). Efficacy of a fabricated customized splint and tendon and nerve gliding exercises for the treatment of carpal tunnel syndrome: a randomized controlled trial. Arch Phys Med Rehabil.

[24] Levine DW, Simmons BP, Koris MJ, Daltroy LH, Hohl GG, Fossel A, Katz JN (1993). A self-administered questionnaire for the assessment of severity of symptoms and functional status in carpal tunnel syndrome. J Bone Joint Surg.

[25] Preston DC, Shapiro BE. Electromyography and neuromuscular disorders: clinical-Electrophysiologic correlations (expert consult - online): Elsevier Health Sciences; 2012.https://www.elsevier.com/books/electromyography-and-neuromuscular-disorders/9781455726721

[26] Kimura J (1979). The carpal tunnel syndrome: localization of conduction abnormalities within the distal segment of the median nerve. Brain.

[27] Johnson E, Kukla R, Wongsam P, Piedmont A (1981). Sensory latencies to the ring finger: normal values and relation to carpal tunnel syndrome. Arch Phys Med Rehabil.

[28] Buschbacher RM (1999). Ulnar nerve motor conduction to the abductor DIGITI MINIMI1. Am J Phys Med Rehabil.

[29] O’Neill John M.D. (2008). Introduction to Musculoskeletal Ultrasound. Musculoskeletal Ultrasound.

[30] DeLong Elizabeth R., DeLong David M., Clarke-Pearson Daniel L. (1988). Comparing the Areas under Two or More Correlated Receiver Operating Characteristic Curves: A Nonparametric Approach. Biometrics.

[31] Buchberger W, Schön G, Strasser K, Jungwirth W (1991). High-resolution ultrasonography of the carpal tunnel. J Ultrasound Med.

[32] Mondelli M, Filippou G, Gallo A, Frediani B (2008). Diagnostic utility of ultrasonography versus nerve conduction studies in mild carpal tunnel syndrome. Arthritis Care Res.

[33] Cartwright MS, Hobson-Webb LD, Boon AJ, Alter KE, Hunt CH, Flores VH, Werner RA, Shook SJ, Thomas TD, Primack SJ (2012). Evidence-based guideline: neuromuscular ultrasound for the diagnosis of carpal tunnel syndrome. Muscle Nerve.

[34] Duncan I, Sullivan P, Lomas F (1999). Sonography in the diagnosis of carpal tunnel syndrome. AJR Am J Roentgenol.

[35] Mallouhi A, Pültzl P, Trieb T, Piza H, Bodner G (2006). Predictors of carpal tunnel syndrome: accuracy of gray-scale and color Doppler sonography. Am J Roentgenol.

[36] Swen W, Jacobs J, Bussemaker F, De Waard J, Bijlsma J (2001). Carpal tunnel sonography by the rheumatologist versus nerve conduction study by the neurologist. J Rheumatol.

[37] Zaidman CM, Al-Lozi M, Pestronk A (2009). Peripheral nerve size in normals and patients with polyneuropathy: an ultrasound study. Muscle Nerve.

[38] Won SJ, Kim BJ, Park KS, Yoon JS, Choi H (2013). Reference values for nerve ultrasonography in the upper extremity. Muscle Nerve.

[39] Altinok T, Baysal O, Karakas H, Sıgırcı A, Alkan A, Kayhan A, Yologlu S (2004). Ultrasonographic assessment of mild and moderate idiopathic carpal tunnel syndrome. Clin Radiol.

[40] Chen S-F, Lu C-H, Huang C-R, Chuang Y-C, Tsai N-W, Chang C-C, Chang W-N: Ultrasonographic median nerve cross-section areas measured by 8-point" inching test" for idiopathic carpal tunnel syndrome: a correlation of nerve conduction study severity and duration of clinical symptoms. BMC Med Imaging 2011, 11(1):1. 10.1186/1471-2342-11-22 PMC3292452 22189264

[41] Klauser AS, Halpern EJ, De Zordo T, Feuchtner GM, Arora R, Gruber J, Martinoli C, Löscher WN (2009). Carpal tunnel syndrome assessment with US: value of additional cross-sectional area measurements of the median nerve in patients versus healthy volunteers. Radiology.

[42] Chen Y-T, Williams L, Zak MJ, Fredericson M (2016). Review of ultrasonography in the diagnosis of carpal tunnel syndrome and a proposed scanning protocol. J Ultrasound Med.

[43] Klauser AS, Ellah MMA, Halpern EJ, Siedentopf C, Auer T, Eberle G, Bellmann-Weiler R, Kremser C, Sojer M, Löscher WN (2015). Sonographic cross-sectional area measurement in carpal tunnel syndrome patients: can delta and ratio calculations predict severity compared to nerve conduction studies?. Eur Radiol.

